# Pressure ulcer related pain in community populations: a prevalence survey

**DOI:** 10.1186/1472-6955-13-16

**Published:** 2014-06-21

**Authors:** Elizabeth McGinnis, Michelle Briggs, Michelle Collinson, Lyn Wilson, Carol Dealey, Julia Brown, Susanne Coleman, Nikki Stubbs, Rebecca Stevenson, E Andrea Nelson, Jane Nixon

**Affiliations:** 1 Leeds Teaching Hospitals NHS Trust, Leeds, UK; 2 Leeds Metropolitan University, Leeds, UK; 3 Clinical Trials Research Unit Leeds, Leeds, UK; 4 University of Birmingham, Birmingham, UK; 5 Leeds Community Healthcare NHS Trust, Leeds, UK; 6 University of Leeds, Leeds, UK

**Keywords:** Pressure Ulcer, Pain, Prevalence, Community

## Abstract

**Background:**

Pressure ulcers are costly to the healthcare provider and can have a major impact on patient’s quality of life. One of the most distressing symptoms reported is pain. There is very little published data on the prevalence and details of pain experienced by patients with pressure ulcers, particularly in community populations. The study was conducted in two community NHS sites in the North of England.

**Methods:**

The aim was to estimate the prevalence of pressure area related pain within a community population. We also explored the type and severity of the pain and its association with pressure ulcer classification. A cross-sectional survey was performed of community nurses caseloads to identify adult patients with pressure ulcers and associated pain. Consenting patients then had a full pain assessment and verification of pressure ulcer grade.

**Results:**

A total of 287 patients were identified with pressure ulcers (0.51 per 1000 adult population). Of the 176 patients who were asked, 133 (75.6%) reported pain. 37 patients consented to a detailed pain assessment. Painful pressure ulcers of all grades and on nearly all body sites were identified. Pain intensity was not related to number or severity of pressure ulcer. Both inflammatory and neuropathic pain were reported at all body sites however the proportion of neuropathic pain was greater in pressure ulcers on lower limbs.

**Conclusions:**

This study has identified the extent and type of pain suffered by community patients with pressure ulcers and indicates the need for systematic and regular pain assessment and treatment.

## Background

A pressure ulcer (PU) is described as a “localised injury to the skin and/or underlying tissue usually over a bony prominence, as a result of pressure, or pressure in combination with shear. A number of contributing or confounding factors are also associated with pressure ulcers; the significance of these factors is yet to be elucidated” [1].

PUs are categorised according to the level of tissue damage. Although many different grading scales exist, an international classification was published in 2009 [1] and includes Category 1 as non-blanching erythema of intact skin, Category 2 as [2] partial thickness dermal loss or blister, Category 3 as full thickness dermal loss with or without a cavity or slough, Category 4 as full thickness dermal loss exposing underlying structures such as muscle, tendon or bone. Pressure ulcers most commonly occur when a person is bedfast, chairfast or spend most of their time in a bed or chair [3]. Body sites where PUs usually occur are bony prominences such as the sacrum, ischeal tuberosities, hips and heels [4] where there is little soft tissue, in particular subcutaneous fat, to provide padding.

Systematic review evidence indicates that pressure ulcers result in significant suffering and morbidity to patients [5]. They are costly to the healthcare provider and in the UK costs to the health and social care system are estimated as £1.77 billion per year [6] and a significant burden to healthcare organisation internationally [2,7]. Patients with PUs are found in all healthcare settings; reports of prevalence and incidence rates vary according to the case mix of the population, the study design and the classification scales used. A review by Garcia [8] identified 9 publications of international origin that reported incidence or prevalence of PUs in home care settings. Incidence rates cited from data base reviews range from 4.5% in Japan [9] to 6.3% in the USA [10], the range of prevalence was 1.3% in the USA [11] to 19.1% in Brazil [12]. Prevalence in UK populations has found to range from 0.74/1000 population [13] to 0.51/1000 population [14].

The impacts of PUs on patients include pain, depression, local infection, osteomyelitis, anaemia, sepsis, gangrene and death [15]. In a systematic review of the quality of life (QOL) literature Gorecki et al. [5] identified that pain was reported by patients as their most troublesome symptom impacting on QOL. A second systematic review by Gorecki et al. [16] aimed to understand the nature of PU pain and map causal pathways from the patients’ perspective. The review identified that PU pain can be debilitating, reducing the individual’s ability to participate in physical and social activities, assume comfortable positions, move, walk, and undergo rehabilitation [16]. People with PUs describe their experience as “endless pain” characterised by constant presence, needing to keep still and describing pain due to equipment and treatments [5,17].

Experience of local pain or discomfort at a potential PU body site may also be a precursor to pressure damage and patients have reported that in their view it was a precursor to PU development [5]. A number of cohort studies have assessed the relationship of potential risk factors to PU development and key variables have emerged including mobility, factors affecting tissue perfusion and skin condition [18]. The assessment of “skin condition” requires clinicians to recognise certain skin changes which may be indicative of pressure damage e.g. local indurations, oedema, localised pain and warmth [19]. These skin change indicators are especially important for people with darkly pigmented skin where non blanching erythema may be missed [20] Although patients are known to report pain, this does not always prompt action and many healthcare professionals dismiss patients’ reports of pain [5,16,21]. This lack of recognition and attention to PU pain may be attributed, in part, to the fact that the role of pain in PU development and treatment is poorly understood by health professionals.

Reviews of the epidemiological literature carried out by Girouard et al. 2008 and Pieper et al. [8,22] identify eight studies reporting the prevalence of pain associated with PUs (sample sizes from 20 to 186 patients) , in hospital, community and palliative care settings. PU pain prevalence estimates were 37% and 66% in the two largest studies with more than 100 patients. Limitations in the literature include the use of non-validated measures of pain (including nurse assessed pain outcomes), small sample sizes, and an absence of studies which report the dominant type of pain. There are essentially two types of pain: nociceptive pain resulting from the inflammatory response, and neuropathic pain occurring as a result of nerve damage or tissue ischaemia [23]. Understanding the characteristics of pain is important as successful pain management depends upon using interventions which address the cause(s) of the pain. A further problem with research in the field is that pain reports are often limited to Category 2 and above PUs [5,8,16,22]. Pain associated with Category 1 PUs is not reported in most studies, nor is the presence of pain on ‘pressure areas’ despite patient reports that pain at ‘pressure areas’ preceded PU development [5].

In summary, qualitative evidence identifies pain as an important issue for patients preceding PU development and in PU management. Previous epidemiological research has focused on patients with existing PUs and a limitation of the literature is the lack of evidence relating to the extent of pain preceding PU development, the extent of pain associated with Category 1 PUs (the most prevalent PU Category) and the type of pain (i.e. inflammatory or neuropathic). This study aims to determine the extent of the problem in community populations.

There is a need to develop methods to assess localised pain in pressure areas, including intact skin and Category 1. In order to describe pain in patient populations with and without PUs four definitions were defined as follows:

1. Pressure area: a body site where PUs commonly develop e.g. sacrum, heels

2. Pressure area related pain: pain on a pressure area (see 1)

3. Pressure ulcer related pain: pain on a body site with an observable PU

4. *Unattributed* pressure area related pain: pain reported by the patient to be due to pressure on a pressure area (see 1) but exact body site not specified.

A study by Briggs et al. [24] reported estimates of prevalence of such pain in hospital populations; they found an overall *unattributed* pressure area related pain prevalence of 16.3% which included a 12.6% *unattributed* pressure area related pain prevalence in patients with no PUs and a 42.9% *unattributed* pressure area related pain prevalence in patients with PUs in hospital populations. It is not known whether the prevalence of pressure area related and PU pain is similar in long term care settings such as patients’ homes and care homes.

The current study investigates the prevalence of PU pain in community populations. Hospital populations are more accessible; the previous study [24] used detailed pain questions with all patients whether they had a pressure ulcer or not. Due to the practical limitations (time to visit widespread geographical locations simultaneously) of the current study, the detailed pain questions were only asked of patients who had a PU and therefore has been reported separately.

This study is part of a suite of 6 studies which comprise the Pressure UlceR Programme of ReSEarch (PURPOSE), funded by the National Institute for Health Research (NIHR,) which aims to reduce the impact of PUs on patients through improved risk assessment and the development of measures to capture patient reported outcomes.

The primary aim of this study was to

● To estimate the prevalence of PU related pain within a community population

The secondary objectives were

● To assess type and severity of PU pain

● To explore the association between pain and PU classification

## Methods

### Study design

We undertook a cross sectional study in 2 community NHS Sites in the north of England to establish PU pain prevalence. Site 1 has a population of 259,536 of people living in urban areas [25]. Site 2 has a population of 307,190 of people [26] with the majority living in rural areas. Community NHS sites include patients in their own homes, intermediate or long term care facilities where some or all of the care is provided by the NHS. The pain survey questions were nested into the routine annual PU prevalence audits undertaken in the participating NHS Trusts. The results of the PU prevalence audits have been published by Stevenson et al. [14]. In addition to the routine PU audit data, patients were asked two questions relating to PU pain by community registered nurses, who were trained in the data collection process, to establish PU pain prevalence. The benefit of using the attending community nursing teams was the ability to screen all potential eligible patients, whilst also minimising burden upon vulnerable patients. These advantages outweighed the impact of multiple assessors upon reliability of data.

### The questions were

1. Do you currently have any pain, soreness, or discomfort, either all the time or on and off in any areas exposed to pressure (e.g. sacrum, buttocks, heels)?

2. Do you think your pain, soreness, or discomfort is related to either your PU or pressure/rubbing due to being in bed/chair?

These questions were adapted from the case screening questions used in a large postal survey of pain prevalence in the UK [27].

Where pain was identified, consenting patients had a detailed pain and skin assessment including pain severity, type and Grade of PU by the Clinical Research Nurse.

### Eligibility criteria

Patients over 18 years old and on community nursing caseloads were eligible to be asked the two pain questions when they had a PU assessed as a Grade 1,2,3,4 or Unstagable [28] and were considered well and able to report the presence or absence of localised pain. Paediatric, obstetric and psychiatric patients were excluded. Patients were also excluded where it was considered ethically or clinically inappropriate by the community nursing team, for example, those where death was imminent. Patients who replied ‘yes’ to both pain questions were then eligible for the detailed pain assessment.

The Clinical Research Nurses were trained in study procedures including pain assessment and skin assessments by the Clinical Co-ordinator (LW) and the site Tissue Viability Nurse Specialist. No formal inter-rater reliability assessment was undertaken since previous research has demonstrated high levels of agreement between specialist nurses and clinical research nurses in skin assessment and PU classification [29].

### Data collection

Standard community practice for the PU prevalence audit was used to assess and record data to ensure data capture for the total population. The two sites applied different eligibility criteria for PU case finding. Site 1 assessed all patients on the community nursing caseload, patients in residential homes, rehabilitation units, specialist palliative care units and all nursing homes in the locality. Patients in site 1 were assessed whether they were known to have a pressure ulcer or not. Site 2 assessed only patients on the community nursing caseload in the locality who were known to have an existing PU. Anonymised individual patient data were recorded by community nurses who were trained in the data collection process. Data recorded included location, date of birth, gender, height, weight, mobility, risk assessment scale (as per local policy) and PU classification by skin site using the EPUAP classifications [28]. Unstagable PUs were recorded as Grade U. Where another skin condition or chronic wound was present on any of the key anatomical skin sites (for example leg ulcer encroaching on a heel area) these were also recorded.

The original (1998) EPUAP classification was used as this was usual clinical practice in both community sites at the time of the routine PU audits.

**Figure 1:**
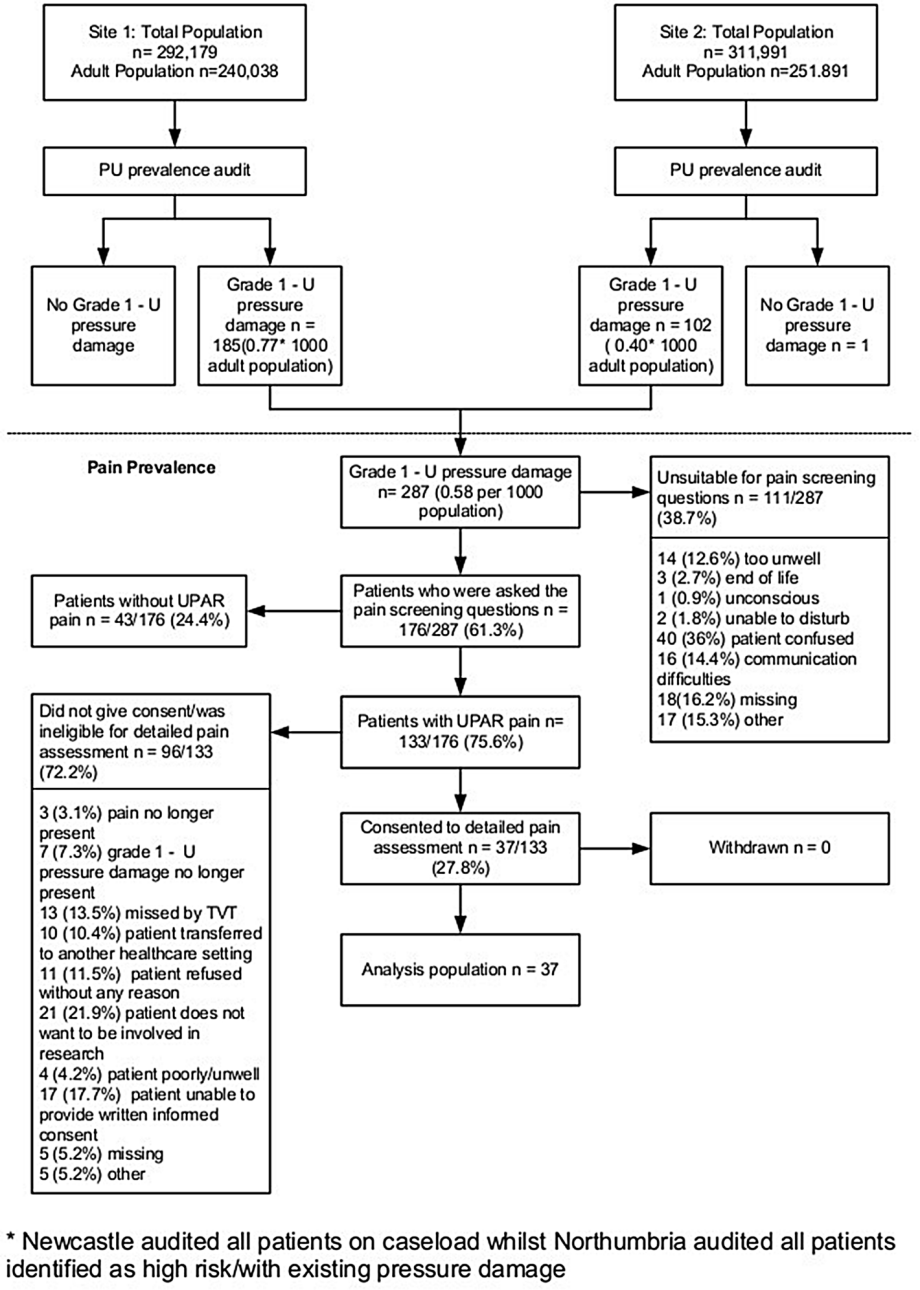
Participant flow.

Where patients were assessed as unsuitable for the pain screening question this was recorded along with the reasons for ineligibility (see Figure 1). Patients assessed as able and therefore eligible were asked the 2 pain screening questions. Patients who answered yes to both questions were then considered for recruitment to the detailed pain study. Assenting patients were seen in their usual place of residence by a Clinical Research Nurse who explained the pain study and gained written or witnessed verbal informed consent. The Clinical Research Nurse undertook a full pain assessment and verification of skin status and PU grade. Pain was assessed by asking patients to: report the pain intensity (for most severe pain over the past week) for all pressure area sites using a numerical rating scale of 0–10 [30,31]; identify their most painful torso and limb skin sites and these were assessed using the Leeds Assessment Neuropathic Symptoms and Signs (LANSS) Pain Scale [30].

The LANSS Scale is a clinically validated tool which allows assessment of neuropathic and inflammatory pain. It consists of a brief clinical assessment and is easy to score in the clinical setting. The questionnaire contains 5 symptom items and 2 clinical sensory testing items associated with neuropathic pain. The two skin sites assessed using the LANSS included the most painful skin site located on the torso (e.g. sacrum, buttocks, ischial tuberosities, hips) and the most painful site located on a limb (e.g. heels, elbows) as reported by the patient. Skin assessments undertaken by the community nurse for the PU prevalence audit were verified through nursing records or clinical assessment by the research nurse.

All data returned to the Clinical Trials Research Unit for data processing was anonymous.

### Analysis

Data were entered into a MACRO database; range and consistency data checks were carried out to assess accuracy of the data. Descriptive statistics were produced, no inferential statistical testing were planned or undertaken. Percentages were calculated using the total number of patients from the relevant population as the denominator (i.e. including all patients with missing data for that variable). All analyses were carried out using SAS software. All percentages were rounded to 1 decimal place. Means, medians, standard deviations and ranges were summarised to one more decimal place than the data collected. The prevalence of pressure ulceration in the population was calculated as a percentage as was the overall proportion of patients reporting localised PU pain.

### Ethical approval

The study was approved by the Leeds West Research Ethics Committee (REC) prior to data collection.

## Results

Site 1 collected data between 8th February and 2nd April 2010 and Site 2 between 12th April and 7th May 2010. Figure 1 details the flow of participants through each stage of the process.

The two community NHS Trusts identified 287 patients with Grade 1-4/Unstagable pressure damage. The case finding methods resulted in differing prevalence rates. In site 1, 1680 patients were assessed and of these 185 patients were assessed as having a pressure ulcer Grade ≥1, a prevalence rate of 0.77 per 1000 (185/240038)*1000 adults). In Site 2 102 patients were identified from the community nursing caseloads and assessed as having a Grade ≥1 pressure ulcer, a prevalence rate of 0.40 per 1000 ((102/251891)*1000 adults).

The mean age of patients with pressure ulcers was 77.8 years (SD 13.44, range 23–106) with just over a third men 34.8% (100/287), 84.3% (242/287) were assessed as ‘at risk’ on either the Waterlow Score or Braden Scale and only 1.4% (4/287) were non Caucasian (Table 1).

**Table 1:** Summary of demographics for total PU, pain and detailed assessment populations

	**Total community PU prevalence**	**Pain prevalence population**	**Detailed pain assessment population**
Total Population	287	176	37
Age (in years):			
Median	81.0	79.0	75.0
Mean (SD)	77.8 (13.44)	76.2 (13.27)	72.6 (15.31)
Range	23.0,106.0	23.0, 99.0	23.0, 98.0
Male	100 (34.8%)	71 (40.3%)	9 (24.3%)
n ‘at risk’ per RAS:			
Waterlow	38/38 (100%)	16/16 (100%)	
Braden	213/242(88.0%)	132/156 (84.6%)	25/37 (67.6%)
Non Caucasian	4 (1.4%)	2 (1.1%)	0 (0%)
Place of assessment:			
Own home	134 (46.7%)	108 (61.4%)	26 (70.3%)
Nursing home	98 (34.1%)	44 (25.0%)	6 (16.2%)
Residential home	36 (12.5%)	10 (5.7%)	3 (8.1%)
Rehabilitation unit	12 (4.2%)	9 (5.1%)	1 (2.7%)
Specialist Palliative Care Unit	5 (1.7%)	4 (2.3%)	1 (2.7%)
Missing	2 (0.7%)	1 (0.6%)	0 (0.0%)
Total number of PUs	440	285	54
Number PU per patient			
Median	1.0	1.0	1.0
Mean (SD)	1.5 (0.83)	1.6 (0.88)	1.5 (0.65)
Range	1.0, 5.0	1.0, 5.0	1.0, 3.0
Grade of PUs reported			
Grade 1	155 (35.2%)	87 (30.5%)	20 (37.0%)
Grade 2	177 (40.2%)	118 (41.4%)	17 (31.5%)
Grade 3	63 (14.3%)	45 (15.8%)	8 (14.8%)
Grade 4	32 (7.3%)	25 (8.8%)	5 (9.3%)
Unstageable	13 (3.0%)	10 (3.5%)	4 (7.4%)

RAS = Risk Assessment Scale.

The 287 participants were reported to have 440 PUs (mean 1.5 per patient, SD 0.83, range 1–5). Approximately a third of PUs (155/440, 35.2%) were Grade 1, 40.2% (177/440) were Grade 2 and 24.5% (108/440) were severe PUs.

### Primary aim

Two hundred and eighty seven patients with PUs were identified and of these 176 (61.3%) were asked the pain screening questions; Figure 1 gives reasons why the remaining patients were unsuitable. The prevalence of pressure ulcer related pain in the population of patients with PUs who were asked the pain screening question was 75.6% (133/176).

Demographic details for the three populations (all patients with PUs, all those who reported pain and those who consented to the detailed pain assessment) are given in Table 1. Data on the number of PUs per patient and the severity of their ulcers has been compared for those who did and did not report pain. The information on those with no pain is taken from the community nurses data and was not confirmed by the clinical research nurses as these patients were out with the study population. This is presented in Table 2. This shows similar severity and numbers of PUs per patient for those with and without pain.

**Table 2:** PU characteristics for patients with and without pain

		**Patients without pain**	**Patients with pain**
Number of patients		43	133
Total number of PUs reported		70	215
Number of PUs per patient	Mean (standard deviation)	1.6 (0.90)	1.6 (0.88)
	Median number of PUs	1.0	1.0
	Range	(1.0, 5.0)	(1.0, 4.0)
	IQR	(1.0, 2.0)	(1.0, 2.0)
Grade	1	23 (32.9%)	64 (29.8%)
	2	22 (31.4%)	96 (44.7%)
	3	10 (14.3%)	35 (16.3%)
	4	13 (18.6%)	12 (5.6%)
	Unstagable	2 (2.9%)	8 (3.7%)

### Secondary objectives

Of the 133 patients with *unattributed* pressure area related pain, 96 were not able or declined to participate in the full pain assessment (see Figure 1). Therefore, the analysis population of eligible patients with *unattributed* pressure area related pain who consented to the detailed pain assessment was 27.8% (n = 37/133) of the population reporting pain.

The mean age of these 37 patients was 72.6 years (SD 15.31; range 23–98), most (70.3%) patients were assessed in their own homes, the remainder were assessed in residential or nursing homes, rehabilitation or palliative care units. Twenty eight patients (75.7%) were female and all were white British ethnic origin (see Table 1).

### Pain and pressure ulcer classification

Pain was reported by patients with all grades of pressure ulcers. It was reported in all but one Grade 1 PU with most of the painful PU being at the sacrum, buttocks and heels. Table 3 show the number of reports of pain for each grade of PU.

**Table 3:** Detailed pain assessment, number of times pain reported by skin classification

	**Yes N (%)**	**No N (%)**	**Total N (%)**
Normal skin	0 (0)	427 (100)	427(100)
Grade 1	19 (95.0)	1 (5.0)	20 (100.0)
Grade 2	17 (100.0)	0 (0.0)	17 (100.0)
Grade 3	8 (100.0)	0 (0.0)	8 (100.0)
Grade 4	5 (100.0)	0 (0.0)	5 (100.0)
Unstagable	4 (100.0)	0 (0.0)	4 (100.0)
**Total**	53 (11.0)	428 (89.0)	481 (100.0)

### Type of pain, severity of pressure ulcer and body site

**Figure 2:**
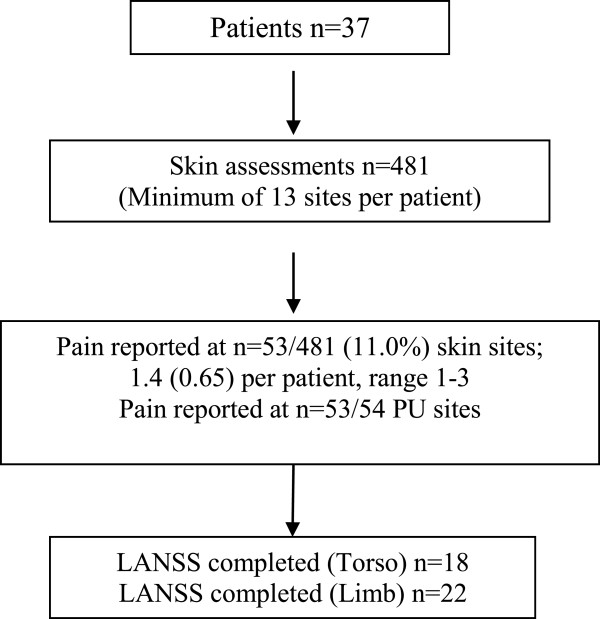
Flow diagram of detailed pain and skin assessments.

A total of 481 skin sites were assessed (see Figure 2), including 427 skin sites assessed as normal and 54 PUs (mean 1.5 per patient; SD 0.65; range 1–3). Approximately a third of PUs were Grade 1 (37.0%; n = 20/54), Grade 2 (31.5%; n = 17/54) and Grade 3/4/U (31.5%; n = 17/54) (see Table 3), with 29 (53.7%) located on a torso skin site and 25 (46.3%) located on a limb skin site.

**Figure 3:**
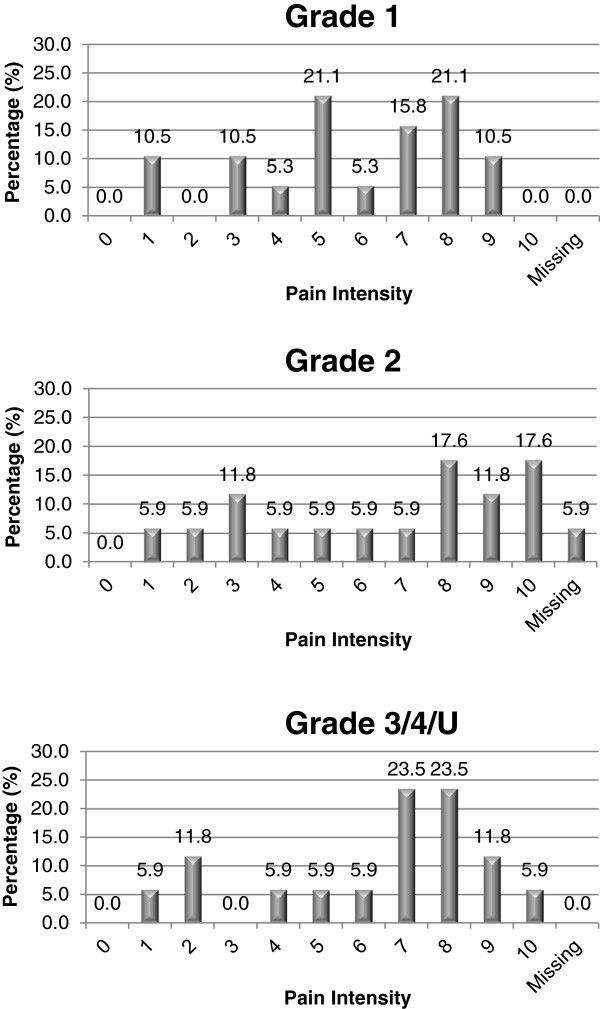
Pain intensity by skin classification.

The 37 patients reported pain on 53/481 (11.0%) skin sites (median 1.0; mean 1.4; SD 0.65; range 1–3). No pressure area related pain was reported on normal skin, whilst patients reported PU pain for 98.1% (n = 53/54) of all PUs (see Table 3). Pain intensity ranged from 1–10, with a mean of 6.4 (SD 2.53) and median of 7.0. There is a slightly skewed distribution of pain intensity with very similar pain levels for each grade of PU (see Figure 3). Thirty one patients identified one skin site for LANSS assessment (n = 19 torso and n = 15 limb) and 6 patients identified both a torso and limb site for LANSS assessment providing a total of 22 torso and 18 limb LANSS assessments. Neuropathic pain was slightly dominant in both torso and limb skin sites, with 54.5% (n = 12/22) of torso PUs and 61.1% (n = 11/18) of limb PUs scoring ≥12 on the LANSS assessment (see Table 4).

**Table 4:** Type of pain by skin classification for the most painful torso and limb areas

**Location**	**Skin classification**	**Nociceptive N (%)**	**Neuropathic N (%)**	**Missing N (%)**	**Total N (%)**
Torso	Grade 1	3 (42.9%)	4 (57.1%)	0 (0.0%)	7 (100.0%)
	Grade 2	3 (33.3%)	6 (66.7%)	0 (0.0%)	9 (100.0%)
	Grade 3	0 (0.0%)	0 (0.0%)	0 (0.0%)	0 ( 0.0%)
	Grade 4	4 (80.0%)	1 (20.0%)	0 (0.0%)	5 (100.0%)
	Unstageable	0 (0.0%)	1 (100.0%)	0 (0.0%)	1 (100.0%)
	**Total**^**#**^	10 (45.5%)	12 (54.5%)	0 (0.0%)	22 (100.0%)
Limb	Grade 1	2 (50.0%)	2 (50.0%)	0 (0.0%)	4 (100.0%)
	Grade 2	1 (20.0%)	4 (80.0%)	0 (0.0%)	5 (100.0%)
	Grade 3	2 (33.3%)	3 (50.0%)	1 (16.7%)	6 (100.0%)
	Grade 4	0 (0.0%)	0 (0.0%)	0 (0.0%)	0 (0.0%)
	Unstageable	1 (33.3%)	2 (66.7%)	0 (0.0%)	3 (100.0%)
	**Total**^**$**^	6 (33.3%)	11 (61.1%)	1 (5.6%)	18 (100.0%)

^#^The denominator here is the number of patients who completed the LANSS for a Torso skin site.

^$^The denominator here is the number of patients who completed the LANSS for a Limb skin site.

## Discussion

This prevalence study was performed in a large community population, using validated measures to assess the presence of pressure area related pain and the intensity and type of pain in people with pressure ulcers. Of the 176 patients with pressure ulcers who were well and able to report pain 75.6% (133) reported *unattributed* pressure area pain. This is similar to results of other smaller studies reporting pressure ulcer pain prevalence ranging from 37% to 66% and is comparable to the prevalence of pain in other chronic wounds in European populations [31,32].

The detailed pain assessment of community patients identified pressure area related pain on all Grades of ulcer. The distribution of pain intensity measured using a 0–10 nominal rating scale was similar for all grades, which is consistent with pain intensity in other disease states, where the severity of illness is not necessarily related to patients’ reports of pain intensity [31]. It is noteworthy that in the community patient population none of the patients reported pressure area related pain on a skin site assessed as normal, whereas in our related hospital pain prevalence patient population 12.6% of patients without PUs reported pressure area related pain [24].

In the community setting neuropathic pain was dominant (54.5% torso and 61.1% limb). The proportion of neuropathic pain observed is similar to the prevalence of neuropathic pain in community leg ulcer patients (43.5%) [31], but greater than the proportion observed in our hospital pain prevalence patient population (29.7% torso and 39.7% limb) [24]. We did not record the duration of the pain or pressure ulcer and this may be related to the type of pain and is an area of further study.

Limitations with the overall pain prevalence estimate of *unattributed* pain are that: skin assessment data was recorded by clinical staff which has inherent limitations [4,29,33] and may have resulted in over or underreporting of pressure ulcers or misclassification of Grade or extent of tissue damage, particularly at Grade 1, which is prone to misclassification [29]; As there were 85 Grade 1 ulcers, however, this source of potential error must be acknowledged. We were not able, due to resource constraints, to have verification of grades by an independent assessor, and we felt it too invasive/burdensome to record skin sites photographically for remote checking. We were not able to record pain treatment and therefore the quality of pain management may differ between settings and impact upon pain reports and the methodology used meant that a significant proportion of patients (38.7%) were not able to participate in the pain prevalence study due to illness (too unwell, end of life, unconscious) or difficulty in assessing (confused or communication difficulty).

The literature review by Peiper et al. [22] did not identify any studies which used validated tools for neuropathic pain assessment. Although the study by Quirono et al. [34] identified pain descriptors for Category 1 PUs, none of the other studies in the review considered Category 1 level damage.

For the 37 patients whose severity of pressure damage was confirmed by the Clinical research Nurse, all but one was experiencing pain related to each of their ulcers. Pain associated with PUs is also described in similar studies of PUs in acute settings identifying 59% pain prevalence [35], with 80% of participants reporting pain for over 1 hour a day [34] and Briggs et al. [24] finding 43% of hospital patients with PUs, reporting pain.

The non-validated data suggests that pain occurs with every grade of PU and that pain and no pain are broadly comparable in terms of proportions at each grade. It suggests that not all Grade 4 PUs are painful whereas grade 1 can be painful. There does not appear to be an association between the number of PUs and pain.

The detailed pain assessments identified that all but one pressure ulcer was painful. This is inconsistent with the unverified data. Explanations for this could be that those who do not report pain may have sensory impairment e.g. spinal cord injury or have sufficient analgesia to mask any pressure ulcer pain. It may be also be due to the small sample size.

The greater proportion of reports of neuropathic compared to inflammatory pain in the lower limbs may be worthy of further exploration. The mechanisms for neuropathic pain are not yet fully understood; it is considered to be a heterogeneous group of conditions that differ not only in aetiology but also location. A review by Jensen et al. [36] states that one of the most common locations for neuropathic pain is the peripheral nerves however this is not informed by studies of pressure ulcers. A study of painful leg ulceration by Briggs et al. [31] identified that 43.5% of patients with lower limb ulceration reported symptoms of neuropathic pain.

A limitation of this study is the small number of patients who consented to the full pain and PU assessment. The data in Table 2 suggests that 43 patients with no pain had pressure damage, however this was not verified as only those who consented to the full assessment has their PU status verified. The study by Briggs et al. [24] found that in hospital patients, pain occurs at every grade including those with no pressure damage and not all skin sites with pressure damage were painful.

Figure 1 identified patients who were screened but found to be unsuitable for the pain questions, of these, half suffer confusion or experienced communication difficulties, and 16% were too ill. In the UK inclusion of patients who lack capacity has to be justified; the criterion is that the research question cannot be answered without them. As this was a first study, exploratory in nature, the inclusion of patients who lacked capacity was not justified. Pressure ulcers are known to occur predominantly in patients who are elderly or debilitate [14], the generalisability of the findings in all PU populations is unknown.

The ability to feel pain and the levels of analgesia were not reported in this study. Reporting both these variables can be subjective, particularly in community settings where access to medical records and prescriptions is difficult. Levels of analgesia can be taken from patient accounts, nursing records or observation of medications in the home however it is likely that the actual medication taken and the level of analgesia achieved vary in individual patients. The ability to feel pain is also dependent on co-morbidities e.g. spinal injury, stroke or diabetes, again records of these may not be reliable in the patients home. This study aimed to explore the association between PUs and pain, the results suggest an association in this sample of patients and it has provided useful baseline data to inform future work.

The findings of this study have clinical relevance for community nurses staff. It is likely that those patients who have PUs will also be experiencing some degree of pain, this needs to be recognised, assessed and management plans implemented. It has been reported that pain can restrict movement [34] which may increase the patients risk of further pressure damage. Nurses also need to pay heed to patients at risk of PUs who report pain as this may be associated with unidentified pressure damage, a skin assessment of the area exposed to pressure should follow.

## Conclusion

This is the first study to report the prevalence of pain associated with PUs in a UK community population, this was found to be 75.6%. Of the patients who consented to a detailed assessment of their PU and associated pain, all reported pain at a PU site. In this small sample, areas of the body with no pressure damage were found not to be painful. Pain intensity was not related to number or severity of ulcers. Both inflammatory and neuropathic pain was identified in both limb and torso PUs, but neuropathic pain was dominant. This study has contributed to knowledge of pressure ulceration in community patients; it will inform future studies. The identification of pain, particularly in the early stages of PU development may be a valuable clinical predictor of further pressure damage.

This article presents independent research commissioned by the National Institute for Health Research (NIHR) under its programme Grants for Applied research funding scheme (RP-PG-0407-10056). The views expressed in this (article/poster/presentation etc.) are those of the author(s) and not necessarily those of the NHS, the NIHR or the Department of Health.

## Competing interests

The authors declare they have no competing interests.

## Authors’ contributions

JN, JB, EAN, MB, EM conceived of the study. EM, MB, MC, LW, CD, JB, SC, NS, RS, EAN and JN have made substantial contributions to the design, acquisition of data and interpretation of data. MC conducted the analysis. EM and JN drafted the manuscript and MB, MC, LW, CD, SC, NS, RS, JB and EAN have been involved in revising it critically for important intellectual content. All authors have given final approval for the version to be published. All read and approved the final manuscript.

## Pre-publication history

The pre-publication history for this paper can be accessed here:

http://www.biomedcentral.com/1472-6955/13/16/prepub
